# Taste Changes after Bariatric Surgery: a Systematic Review

**DOI:** 10.1007/s11695-018-3420-8

**Published:** 2018-07-31

**Authors:** Kasim Ahmed, Nicholas Penney, Ara Darzi, Sanjay Purkayastha

**Affiliations:** 1 Department of Surgery and Cancer, Imperial College London, London, UK; 2 Imperial Weight Centre, St Mary’s Hospital, London, UK

**Keywords:** Bariatric surgery, Obesity, Taste, Olfaction, Gustation

## Abstract

**Background:**

Alterations in taste perception and preferences may contribute to dietary changes and subsequent weight loss following bariatric surgery.

**Methods:**

A systematic search was performed to identify all articles investigating gustation, olfaction, and sensory perception in both animal and human studies following bariatric procedures.

**Results:**

Two hundred fifty-five articles were identified after database searches, bibliography inclusions and deduplication. Sixty-one articles were included. These articles provide evidence supporting changes in taste perception and hedonic taste following bariatric procedures. Taste sensitivity to sweet and fatty stimuli appears to increase post-operatively. Additionally, patients also have a reduced hedonic response to these stimuli.

**Conclusions:**

Available evidence suggests that there is a change in taste perception following bariatric procedures, which may contribute to long-term maintenance of weight loss following surgery.

**Electronic supplementary material:**

The online version of this article (10.1007/s11695-018-3420-8) contains supplementary material, which is available to authorized users.

## Introduction

Over recent decades, obesity has evolved into a global epidemic. The latest WHO update estimates that 13% of the global adult population is clinically obese, with a further 39% overweight [1]. Bariatric procedures, such as Roux-en-Y gastric bypass (RYGB) and vertical sleeve gastrectomy (VSG), have emerged as the most effective treatment for obesity, with superior long-term weight loss compared to other measures and proving highly effective for the treatment of metabolic comorbidities such as type 2 diabetes mellitus [2, 3].

These results are due to a number of complex mechanisms that we are only beginning to fully understand, including the BRAVE effects of bypass surgery (bile flow alteration, restriction of stomach size, altered anatomy/flow of nutrients, vagal manipulation and enteric gut and adipose hormone modulation) and the SLIMMER effects of bile acids (satiety, lipid and cholesterol metabolism, incretins and glucose homeostasis, energy metabolism, gut microbiota and endoplasmic reticulum stress) [4, 5]. Additionally, patients’ taste and food preferences appear to change after surgery. Hence, this systematic review aims to assess the current understanding of changes that occur within the gustatory system following bariatric procedures.

The gustatory system is a complex series of pathways encompassing the detection of taste stimuli, the cognitive processes of stimulus identification, and the responses via dopaminergic pathways. Behaviourally, taste can be separated into five attributes—onset, intensity, quality, hedonics (reward) and oral localisation, as well as being characterised by the five classical taste characteristics (bitter, salty, sweet, sour and umami) [6]. Biologically, sensory inputs are detected by gustatory cells via activation of specific gustatory receptors, which differ in structure based on the stimulus. Bitter and umami taste stimuli activate G-protein coupled receptors, while amino acids, sugars and artificial sweeteners act on heteromeric protein channels, and salty and sour stimuli are thought to act via direct interaction with ion channels. Additionally, the interplay between the olfactory and gustatory systems is well established. The sense of smell can be activated orthonasally (through inhalation) or retronasally (from the pharynx). Retronasal scent provides an additional boost to taste—flavours are “smelt” from within the pharynx, enhancing flavour perception [7, 8].

Obesity has long been known to cause alterations in gustatory perception—both sensory and hedonic. Obesity has been described as a state in which dopamine signalling is altered. Wang et al. (2001) found a decrease in dopamine receptors (D_2_) in the striatum of an obese individual undergoing PET scanning, which was negatively correlated with BMI, suggesting that increased adiposity causes a long-term downregulation of dopamine receptors [9, 10]. Furthermore, studies have shown a decreased response to taste stimuli in animal models of obesity in both behavioural responses to stimuli and also through in vitro cellular studies [11–13]. Obese animal models have a decreased ability to detect lower concentrations of lipid solutions, suggesting that high-fat, obesity-inducing diets decrease lipid sensitivity by increasing the oral threshold for detection [12] and further a study noted on a cellular level that obese mice had a lower Ca^2+^ response to sweet and bitter stimuli, as well as displaying less evasive behaviour to bitter responses [11]. Similarly, candidates for bariatric surgery decreased salivary response to sour stimuli and a lower level of salivatory habituation [14], and obese children and adolescents have a significantly decreased sensitivity and perceived intensity across multiple taste domains when compared to normal weight controls [15].

Following bariatric surgery, patients are known to change their food preferences and these changes are dependent on the type of procedure [16]. While the restrictive nature of these procedures would be expected to cause a decrease in portion sizes, a change in preferences suggests a change in underlying physiological responses to food. This has been theorised as partly due to dumping syndrome after metabolic procedures such as RYGB but may in part be due to an underlying functional change, including a shift of the gut hormone profile towards an anorexic state [17]. These changes are long lasting, with patients followed up to two and a half years post-surgery maintaining an aversion to high-calorie foods [18]. Hedonic hunger (i.e., food seeking behaviour for increased enjoyment) is increased in obese subjects, who attain much higher scores on ‘power of food’ questionnaires signalling an increased role in the presence and the wanting of food in dictating behaviour. Interestingly, this difference is reversed in post-operative bariatric patients, who have a similar index in comparison to non-obese controls, perhaps implicating altered taste in behavioural changes following RYGB [19]. Additionally, changes have been noted in food cravings for sweet, high-carbohydrate and fast foods post-surgically. Patients who had higher cravings before undergoing bariatric procedures were found to have significantly decreased cravings and hedonic responses when matched with normal weight controls post-op, independent of weight loss [20].

Changes to taste have also been reported following treatment for upper gastrointestinal malignancies. Harris et al. (2003) reported 44 of 99 patients acquiring a deficit in taste or in both taste and smell following oesophagectomy or gastrectomy (and an additional patient with an isolated olfactory deficit) [21]. Thirty of these patients recovered totally after 1 year, with the others having a partial deficit at continued follow up. Taste has also been used as a measure of long-term quality of life. Patients who have undergone total gastrectomy are found to have a significantly worse outcome in taste (as well as other parameters such as social functioning, nausea, and vomiting and eating restrictions) contributing to a significantly decreased quality of life [22]. Changes to taste following bypass procedures in oncological patients may also apply to bariatric patients with similar anatomical interventions.

Here, we conduct a systematic review to collate evidence that supports or refutes the hypothesis that bariatric surgery induces a change in taste perception and activation of reward pathways and, if present, whether changes to taste and smell perception contribute positively to weight loss.

Commonly used terms relating to taste within this article are defined in Table 1.

**Table 1:** Definitions of commonly used terms

Term	Definition
Taste acuity	The ability to detect and recognise taste stimuli [23]
Detection threshold	The lowest concentration at which a taste solution can be distinguished from water [23]
Recognition threshold	The lowest concentration at which a taste solution can be correctly identified [23]
Taste sensitivity	The minimum concentration at which a specific taste quality can be perceived [24]
Hedonic taste	The affective component of taste - whether a taste is liked or disliked [6]
Negative allisthaesia	The point at which a pleasant sensation becomes unpleasant following repeated exposure [25]

## Methods

A systematic search of two databases (OViD Medline and Embase) was carried out to identify all articles investigating the effect of bariatric surgery on the gustatory system, up to January 2017. The search was expanded to include smell and olfaction underneath the heading of taste. The search was carried out using Boolean logic to combine search terms under each search heading (‘taste’, ‘smell’ and ‘bariatric surgery’) into a list of as many possible relevant terms, which was then expanded using the features of the search engines to further add to the list (see Supplementary Table 1). Additionally, Medical Subject Heading terms were added to include synonyms of following search terms, identified using PubMed: Taste, ‘Taste Perception’, ‘Taste Sensitivity’, Bariatric Surgery, ‘Gastric Bypass’, ‘Jejunoileal Bypass’. Articles were then integrated into a single list where all duplicated articles were excluded and articles were reviewed based on title and abstract for relevance. Exclusion criteria included articles assessing non-bariatric procedures (e.g. gastrectomy secondary to gastric cancer); articles assessing obese patients, or patients pre-surgically without post-surgical follow up; articles assessing satiety or hunger instead of gustation; non-English language articles; review articles; editorials; erratum and letters. Successful articles were then reviewed based on the full body of the article for inclusion. Additional articles were identified through a manual search of the bibliographies of retrieved full-text papers.

## Results

**Fig. 1:**
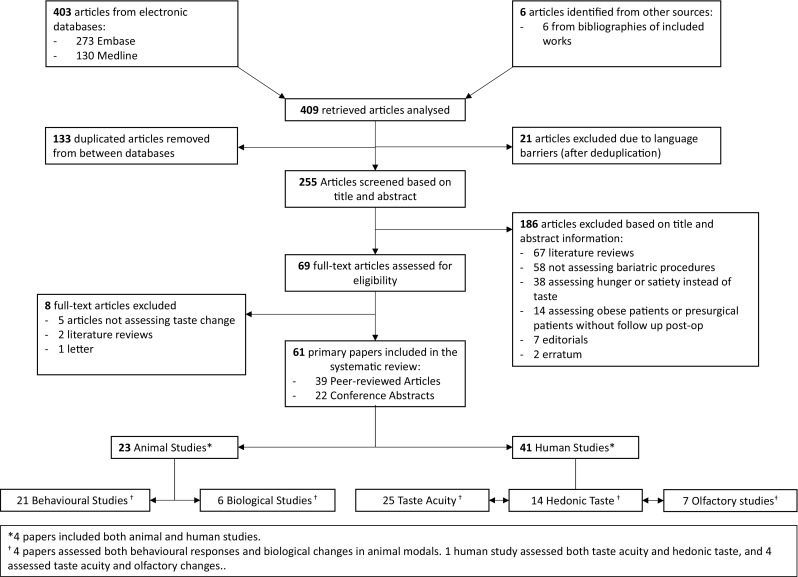
PRISMA flow diagram showing the results of the search and the exclusion process

Searches of Medline and Embase databases yielded 409 articles (of which 133 were duplicates and 21 non-English language); a further 6 articles were included from bibliography searches, resulting in 255 articles from the primary search. Of these, a further 147 were excluded through analysis of the title and abstract (under the criteria mentioned above), resulting in 69 articles selected for full-text review. Further articles were excluded after reading the full text (*n* = 8), and the remaining papers (*n* = 61) were included in the systematic review (see Fig. 1).

Research into the effects of bariatric surgery on the gustatory system can be categorised based on the nature of the studies: animal models vs humans. Studies using animal models generally observe behavioural changes in response to food stimuli or study neurological, biological or hormonal changes post-operatively. Human studies are also broadly split into two categories: those studying taste acuity and sensation and research into the hedonic responses to taste stimuli. Human studies using experimental data had fewer participants, with more recent studies providing some exceptions (e.g. Altun et al., 2016 *n* = 52), while questionnaire studies were able to obtain greater participant numbers. Owing to the heterogeneous nature of existing data, this review has been structured as a narrative covering each of these domains, with an analysis of the data synthesis provided in each section accordingly.

## Discussion

### Behaviour Changes in Animal Models

Numerous studies have been carried out on animal models of bariatric surgery to determine changes in behavioural patterns and preferences to certain foods. In 1981, Sclafani et al. used a two-bottle choice test to determine changes in food preference in normal-weight rats post-Jejunoileal Bypass (JIB) against sham-operated rats. In this early study, bypass surgery induced a strong and persistent aversion to sweet-tasting solutions [26]. A series of more comprehensive experiments were later conducted to investigate taste-oriented changes in rat models of JIB, giving insight to the role of bariatric surgery in these aversive behaviours. Rats that underwent JIB developed a conditioned taste aversion response when immediately exposed to a sweet-tasting solution. However, less aversion was noted when rats were pre-exposed. This aversion gradually diminished after reversal of surgery suggesting an induced change in taste perception [27]. JIB rats also had a decreased preference to saccharin-flavoured foods and proportionally less intake of sweet solids than non-sweetened standard chow.

Since then, studies into taste behaviour have mostly been measured by preferential behaviour on exposure to different stimuli at different concentrations. Models for this are related to time exposure to the stimulus—either brief access (in seconds) or longer access (over minutes, hours or days), with measurements taken based on volumes consumed or by assessing the ‘lick responsiveness’ (i.e. the number of tastes taken by the rat). Additionally, comparison studies between different stimuli through two-bottle test exposure allow a measurement of preference in animal models.

Brief access exposure allows a better view of sensory responses to stimuli. This method involves a very short period of exposure (10–20 s) and assesses how receptive to the stimulus each subject is, giving an impression for immediate sensory responses to the stimulus itself. However, tests must be standardised in terms of exposure to foods and fluids in order for results to be replicatable. This method was used by Hajnal et al. (2010), during which brief access was given to fasted pre- and post-operative rats to varying concentrations of different taste stimuli. Preference for sweet stimuli (sucrose, fructose and alanine) was decreased after RYGB, while no changes were found in response to other taste stimuli (NaCl, Citrate and Quinine) [28]. Another study measured preferences after 10 s of exposure to different concentrations of sucrose solution in RYGB and sham-operated rats by recording the number of tastes taken. This showed that post-RYGB rats had a significantly decreased preference to higher concentrations of sucrose compared to controls, with no difference to water intake [29]. These results have been repeated using variations of the same method [30] and have found similar responses to other natural sugars but not to artificial sweeteners [31]. However, contrasting results using a similar brief access test design have also been shown [32]. RYGB rats showed no attenuation in lick responsiveness when compared to sham controls and actually found an increase in appetitive behaviour and consummatory behaviour in some subjects. This may have been due to the nature of the brief access test, as it stimulates an immediate orosensory response without stimulating post-ingestive effects, or it may signify that behavioural and environmental factors play a greater role in food preferences. Furthermore, a previous brief access trial assessing food-deprived behaviour in RYGB rats vs sham controls showed no difference in sample tastes when RYGB rats were food deprived and increased sucrose tasting when not deprived [33].

Longer access testing can give indication to long-term food preferences and food reward values experienced. Studies following a similar structure to those above with longer exposure have found similar results. Le Roux et al. (2011) used 48-h exposure to assess energy intake in solid foods and preferences for higher-calorie liquids using the two-bottle preference test model. RYGB rats had significantly reduced intake of high energy and flavoured alternatives, though increased proportional intake of normal chow (with an overall decrease in food consumption). Comparison of high-fat liquid (intralipid) to water found that while RYGB rats had some initial preference for the high-fat solution, it was significantly less than sham-operated controls. By day 200, there was no preference shown for higher concentrations of the lipid solution [34]. While these results stand true on long exposure, other studies have found that on brief exposure, there is no significant difference between RYGB rats and controls, suggesting that post-absorptive effects may have a role in decreased fat preference [35]. Additionally, 24-h access to different taste stimuli in post-surgical RYGB models shows lower intake of sucrose at higher concentrations than controls, as well as significantly decreased preference for high-sucrose solutions when offered as part of a two-bottle test. Furthermore, pre-surgical exposure to sucrose solutions causes a significantly higher preference to sucrose post-surgically than in rats without previous exposure (though still lower than controls), suggesting a role of pre-operative diet in changes to behaviour post-operatively [36]. Other studies of long exposure have found similar results in sucrose and lipid preference on 48-h two-bottle preference testing [37]. Moreover, another study also tested the effects of 60-min ‘bursts’ of exposure to sweet, intralipid and high-energy (Ensure) solutions over a 5-day period. RYGB rats were shown to have overall lower consumption than sham-operated controls; however, the first exposure of sucrose was comparable to controls with consumption decreasing with each subsequent exposure. Lower consumption was noted in the high-fat intralipid solution and ensure on-repeat exposure, though ensure was significantly lower even on initial exposure. This suggests that experience post-operatively is required to identify the reduced preference to certain stimuli, as opposed to an immediate orosensory response to the substance being consumed [38]. Other long-exposure studies agree with these findings, suggesting that RYGB rats have a higher preference for lower concentrations of sweet-tasting and high-fat solutions [39–41]. Similar results have been found in which RYGB rats show decreased preference, the results being more pronounced in rats which are exposed pre-operatively to lower-fat diets than those exposed to high-fat diets [42].

Additionally, these results translate to other forms of bariatric surgery [43]. Wilson-Perez et al. (2013) postulated that rats post-VSG would have a greater attraction to high-calorie foods, due to the change in demand following a restrictive procedure. However, it was found when comparing RYGB and VSG that rats after each operation had similar aversive responses to high-calorie foods and almost identical profiles in terms of amounts consumed and food preferences. This suggests that VSG may have more of a metabolic impact than initially thought. Another study compared brief access exposure and 24-h two-bottle tests in RYGB and ileal interposition, showing that RYGB reduced preference for higher concentrations while ileal interposition had no effect on taste preference or weight loss [44].

Studies into behaviour based on hedonic sensation show variable results. The use of progressive ratio behavioural tasks provides an insight into changes in the extent at which a subject is willing to perform a task in order to receive a reward. Mathes et al. (2015) measured differences in breakpoints between ensure, intralipid and sucrose-based solutions in RYGB and sham-operated rats that were fed and food-deprived. Surprisingly, RYGB rats showed no difference in preference to ensure, intralipid or sucrose post-operatively when compared to themselves pre-operatively and in some cases showed a higher breakpoint than the sham-operated controls [45]. Similarly, when challenged in an incentive-based runway towards a sweet food reward, RYGB rats were faster than obese rats at reaching the incentive food [30]. While this would indicate that RYGB rats show greater appetitive behaviour towards sweet foods, the fact that, with the allowance for weight loss to stabilise, times were similar to lean controls suggests a correction to motivational barriers induced by obesity. The same study also shows similar findings on brief access testing to others, in which the RYGB cohort had less preference towards high-concentration sweet foods.

Another found increased consumption of alcohol at higher concentrations after RYGB than normal weight controls or obese rats, a finding that corroborates with the above studies showing increased reward-seeking behaviour in RYGB rats. Further study into the subject is needed to verify these results [46].

### Neurobiological and Hormonal Findings in Animal Models

The biological basis of changes associated with obesity and RYGB has also been the subject of much discussion, aiming to determine potential underlying mechanisms for behavioural changes. Neurological studies seek to identify and isolate activity in different regions of the brain in response to taste stimuli. Rat models of obesity show different patterns of stimulation in response to sweet stimuli compared to normal weight controls in the (PBN) [47]. Electrophysiology studies have been used to identify changes in activity of the PBN within the pons in rats pre- and post-RYGB compared to controls. These studies have found differences in the neuronal activity in obese and post-RYGB rats in response to sucrose solutions [28, 48]. Obese rats have a decreased PBN response to lower concentrations of sucrose and strongly increased responses to high concentrations [47]. Conversely, post-operative RYGB rats show neuronal profiles similar to those of lean rats, with increased activation at lower concentrations which plateau at concentrations above 0.5M [28]. These results reflect the behavioural changes displayed post-RYGB. Changes in brain dopamine have also been studied; however, no notable changes were detected in baseline levels of dopamine in RYGB rats compared to sham-operate controls [37].

Imaging has enabled new insights into neurobiological activity. Global changes have been assessed using PET scans. Thanos et al. (2015) found that areas of the midline and right cerebellum were selectively activated in response to reward, as well as activation of the medial PBN and dorsomedial tegmental areas in expectation of taste based reward. This differs greatly from sham-operated control rats, which showed activation of the left cerebellum, retrosplenial area and the right primary visual cortex, suggesting neurological changes in response to the changes induced by RYGB, which affect food choice and preference [49].

Endocrine involvement in the gustatory system has long been postulated, and signalling alterations may help to explain some of the changes found after bariatric procedures. As such, hormonal assays have also been conducted to help further understand the changes relating to taste and behaviour. Gastrointestinal hormones related to hunger and satiety have been studied in animal models of obesity and bariatric surgery. Animal models of RYGB have shown an increase in post-prandial levels of Ghrelin and Peptide-YY, as well as an increase in circulating GLP-1 [34, 36, 41].

Peptide-YY signalling has been implicated in animal models in decreasing responsiveness to bitter-tasting compounds, fat-based solutions and emulsions [50]. Both RYGB and LAGB decrease levels of circulating PYY when fasting and significantly increased secretion after a meal [51–53]. Whether this is a return to a baseline seen in non-obese patients or if it occurs as an isolated change based on RYGB surgery remains dubious, with some suggesting that RYGB causes a shift in resting baseline that is lower than normal-weight control subjects [52]. Changes in PYY secretion have also been found to be specific to bariatric procedures, not induced by dietary weight loss [51]. GLP-1 is actively secreted from within the gut and locally within taste buds, and GLP-1 knockout mice have been shown to have a markedly reduced response to sweet-taste stimuli at lower concentrations. This suggests that GLP-1 may have a localised and systemic role in modulating taste and satiety [17, 54].

Ghrelin has also been reported as having a role in taste perception. Intraperitoneal injections of ghrelin have been found to cause increased consumption of saccharin and an increased preference for sweet foods, suggesting a role of ghrelin in modulating preference for flavours which confer high calorific content [55]. This is further verified by a decrease in sensitivity and preference for lipid tastes shown in ghrelin and ghrelin receptor knockout mice [56]. Obesity causes a significant decrease in fasting levels of circulating ghrelin [57]. RYGB has been found to cause an even greater decrease in circulating ghrelin in response to a glucose meal than lean controls, as well as a lower fasting level of ghrelin [58] and a loss in diurnal variation of ghrelin levels. This is hypothesised to be due to isolation of ghrelin in the duodenum due to the anatomical changes of RYGB and is thought to contribute to long-term weight control. It may also contribute to the changes in taste sensation [59].

### Human Studies: Non-specific Changes in Patients Post-Operatively

Questionnaire studies have proved useful in providing datasets for large numbers of patients. Surveys help to identify the proportion of patients reporting perceived changes in taste post-operatively. While capable of gauging the spread of perceived changes, they are less good at quantifying these perceptions. A sample of 103 RYGB patients found that 73% of patients found a change in the taste of commonly consumed foods, either through heightened or reduced sensation. The most reported change was in the taste of meat, along with a greatest noticeable change in sweet-tasting foods and additional significant changes in salty and sour foods [60, 61]. A comparison between RYGB (*n* = 82) and LAGB (*n* = 28) found that 86% of RYGB patients and 46% of LAGB reported changes in taste, suggesting a further metabolic role following RYGB in the changes perceived, rather than simple physical restriction [62]. A recent survey of 115 patients following LAGB found that 64% of patients reported an increase in sweet taste 6 months post-operatively, which steadily decreased to 58% at 12 months and 34% at 36 months. These changes were reported as having significant correlation with final weight loss outcomes [63].

Studies on patients after VSG have varying results. In an Asian cohort, over a quarter of patients (33/120) reported changes in taste perception, some to specific taste domains (sweet, salty, sour), others to multiple, with most patients reporting an increase in sensitivity, but with some reporting decreased sensation [64]. Where patients reported increased sensation, they also reported a reduced intake of foods under the same domain. Coluzzi et al. found that 75% of patients reported decreased interest in sweet-tasting foods 6 months following surgery; however, only 23% had persisting change at 12 months. Long-lasting changes in taste and decreased interest in fat and alcohol (70% at 24 months) were also found [65]. Additionally, Gero et al. report a decreased preference to various stimuli at 6 months post-operatively—most significantly sweet taste, but some patients also reported a decreased preference to sour, bitter, umami and salty foods [66]. A comparison of changes in both RYGB and VSG found similar results for both procedures (63%). Most patients reported an increase in sweet taste sensitivity, while some also reported changes in sour and salty taste. Patients noticed these changes in association with meats, sweets and fatty foods [67]. Evidence from longitudinal studies into food preferences suggest that VSG patients tend to have a higher proportional intake of fatty and sweet foods in the long term, while RYGB patients have a much higher preference and consumption of fruits and vegetables, though both groups report overall similar energy intake [34, 41]. Furthermore, Van Vuuren et al. (2016) found a significant increase in spicy and fatty taste intensity between 3 and 6 months in 44% of patients (*n* = 50), and an increase in patients’ reported intensity across other taste groups (bitter, sweet, salty) with the exception of metallic taste, suggesting that changes post-operatively may be exacerbated by weight loss. Further smaller studies have also reported similar results [68]. Comparisons between RYGB and VSG find that changes in taste are more common in RYGB; however, they are still present in a significant proportion of patients following VSG. Makaronidis et al. also found that changes in taste following RYGB correlated significantly with increased percentage weight loss (27.8%, *n* = 3, compared to 23.1% *n* = 35 with no taste changes) [69, 70]. Confounding factors including patient selection, procedure selection and questionnaire design may have influenced these findings and further investigations to quantify reported changes are needed. A recent study suggests that patients with the greatest weight loss have a significantly decreased preference to sweet-tasting foods post-operatively [71]. A similar study in subjective taste analysis via questionnaire found an increase in perceived taste intensity over a 6-month post-operative period [72].

### Human Studies: Taste Acuity

Changes in the sensation of taste have been reported in both obese and post-bariatric surgery patients, especially those who have undergone RYGB. An earlier study compared RYGB patients both pre- and post-operatively, using a three drop model testing detection and recognition thresholds of various taste stimuli. While pre-operatively there was no significant difference between patients and controls, postoperatively, there was significant decrease in patient thresholds for bitter and sour taste and a non-significant decrease in sweet and salty taste [73]. Shortly afterwards, Burge et al. [74] found an increase in sensitivity to sucrose stimulation 6 weeks post-operatively, being able to distinguish sweet-tasting stimuli at almost half the concentration than pre-RYGB (mean recognition threshold fell from 0.047 to 0.024 M/l), which was consistent at follow up 3 months post-op. This finding was isolated solely to sweet taste; no change in bitter taste recognition was found in patients following RYGB. Similar results have also been recorded comparing obese groups to normal-weight controls pre- and post-operatively [36]. When exposed to varying sucrose concentrations for 5 s, RYGB patients had significant improvement in sucrose detection against controls, with post-op patients showing a mean detection threshold at 7.8 mM compared to controls 14.0 mM (pre-op detections levels were 10.8 vs 11.0 respectively). These results suggest an increase in threshold to greater than the normal, not simply a return to the baseline. Furthermore, there was a significant increase when comparing pre-operative results to those following RYGB, though small group size (*n* = 9) may be a confounding factor of this study. Further studies have expanded on this experimental model to include other taste domains. A recent comparison between RYGB patients pre- and post-operatively against normal controls in detecting saltiness in soup solutions found no statistical difference in the detection of salty foods post-operatively or compared to controls. However, this study is one of the first of its kind measuring salt as part of a food solution and used a small sample size (*n* = 14) [75]. Comparisons of taste acuity between RYGB and VSG patients suggest RYGB patients have a significantly higher threshold to sourness, translating as a lower sensitivity [76]. Measurements of bitter and umami thresholds have shown that patients have increased sensitivity post-operatively [77] as well as changes in the perceived intensity of fatty solutions [78, 79].

Moreover, taste strips have been used to measure sensitivities as a result of VSG. Holinski et al. (2015) found an increase in taste sensitivity towards bitter and sweet tastes following VSG similar to those changes measured in RYGB patients [80]. Additionally, Altun et al. discuss changes in short- and long-term gustatory sensitivity to a number of stimuli [81]. Their results showed an increase in taste sensitivity across all domains and all patients in sweet and salty and in some patients in response to bitter and sour taste strips. This was significant initially and up to 3 months post-op. These direct measurements of taste change in VSG patients correlate well with animal models, showing comparable results between VSG and RYGB behavioural responses. Conversely, a similarly organised study found no changes in taste sensation or taste discrimination after either RYGB or VSG, though this is postulated to be due to measurements being taken during the post-operative recovery period [82].

Other studies have found varying degrees of changes in sensory taste perception after RYGB. One study found no significant changes to sensory detection post-operatively and found sucrose was perceived to be less sweet post-surgically, as opposed to the increased sensitivity reported in other papers [83]. Another reported no change in sweet, savoury or salty perception after RYGB, instead suggesting that changes in eating behaviour are not due to direct taste acuity but as a result of changes in the reward value of taste stimuli [84, 85].

### Human Studies: Olfactory Changes

Some studies also explore changes in olfactory perception following bariatric surgery.

Makaronidis et al. found that, in addition to changes in taste post-operatively, 37.9% of patients reported an unspecified change in their smell 36 months post-RYGB, and 21.6% following VSG [69]. Likewise, Zerrweck et al. (2015) found that 54% of RYGB patients (*n* = 104) reported an unspecified change in smell following surgery, most notably a change in the smell of fatty, sweet and meaty foods [67].

Olfactory acuity has also been studied using increasing concentrations of smell stimuli, similar to taste acuity tests. The cross-cultural smell identification test, a self-administered, age and gender matched test, was used to assess olfactory dysfunction in pre- and post-operative patients undergoing RYGB with elective cholecystectomy [86]. This study found persistence in smell deficit and the development of absolute olfactory deficit in three patients despite weight loss, suggesting that olfactory dysfunction may be a causative factor in the development of obesity, rather than a consequence of increased BMI. The use of “Sniffin Sticks” may provide a more accurate representation of a patient’s olfactory function, in which increasing concentrations of smell stimuli are introduced to the patient, who are then scored on their smell threshold(T), and ability to discriminate(D) and identify(I) smell stimuli [87]. This method has been used by multiple groups with varying results. A study into long-term changes in VSG patients found a significant increase in all three parameters (T, D and I) with continuous improvement up to 6 months post-operatively to all stimuli [88]. Converse results were found when TDI scores in 15 RYGB and 15 VSG patients were compared pre- and post-operatively. Only an increase in initial sensing threshold in the VSG group was found, with no changes to discrimination and identification in either group post-operatively [89].

Furthermore, olfactory function has been reported to improve significantly in obese patients following RYGB and VSG, returning to a level closer to normal-weight controls. This study also noted that 7 out of 10 patients who had previously been defined as hyposmic returned to normal functioning smell 6 months post-operatively [80]. Another group reported a significant increase in smell detection thresholds in patients following sleeve gastrectomy [82]. These studies suggest a link between changes in the two systems; however, research directly comparing the two is currently limited.

The metabolic role of olfaction has recently been described in animal models. Riera et al. found a metabolic shift in animal models with olfactory hyper- and hyposensitivity [90]. Olfactory knockout mice had increased brown fat conversion and more actively oxidising fatty acids. As a result, these mice were protected against obesity on a high-energy diet relative to controls and were also protected against developing insulin resistance. Conversely, hypersensitive mice were more prone to developing obesity and insulin resistance. These findings suggest that the olfactory system acts in a bidirectional manner to control energy homeostasis in response to sensory and hormonal signals [90]. However, interestingly, these findings are contrary to what is seen in human studies where olfactory desensitisation is associated with obesity and olfactory changes following surgical intervention in patients often include an increase in sensitivity.

### Human Studies: Hedonic Taste

Similar to experiments into taste acuity, taste hedonics and the pleasantness of food stimuli have long been a subject for study in bariatric patients. Since the advent of intestinal bypass, studies have found changes in food preference in bariatric patients and sought to offer explanations for this. Bray et al. were the first to note changes in preference to sweet-tasting solutions in post-operative patients. Post- jejunoileal bypass patients had less preference to higher concentrations of sucrose solution, peaking at 10% sucrose concentration (1 M), when previously, preference continued to increase up to 40% concentration [91]. Rodin et al. sought to isolate whether this effect was secondary to weight loss or surgical intervention, noting no change in taste acuity in patients post-jejunoileal bypass but a similar decrease in preference. Patients were also compared to obese subjects who had lost weight through dietary intervention. Unlike surgically induced weight loss, dietary intervention caused no real change in preferences, even showing an increased preference at certain concentrations [92].

Assessment of functional hedonics can be carried out using reward-based tasks, in which patients are rewarded at intervals with a food-based reward until reaching a breakpoint in activity. Miras et al. performed one such study using a mouse-clicking reward task in two cohorts—one receiving a sweet reward and the other receiving a vegetable reward—and found a 50% reduction of the breakpoint in RYGB patients post-operatively when offered a sweet reward, however no change in breakpoint when offered a vegetable reward. Additionally, no change was found between normal-weight controls and patients pre-operatively, suggesting that surgery has a role in inducing hedonic change as opposed to solely decreased BMI [93]. Studies comparing different surgical procedures have found that not all bariatric procedures produce a change in food reward. A comparison of the effects of repeat exposure on taste preference after RYGB and LAGB found that RYGB patients had experienced an earlier shift from pleasant to unpleasant upon repeat tasting of a sucrose-based solution than pre-operatively, while LAGB did not cause significant change [83]. Unlike LAGB, duodenal switch (DS) was found to have a similar effect on taste hedonics to RYGB. A study into the time for negative alliesthesia to be achieved in DS patients vs obese controls found that sweet-tasting stimuli became unpleasant sooner 3 months post-operatively in DS patients and sooner still at 6 months. The same held true when measuring the point of satiety, at which the stimulus became intolerable; however, due to small sample size, these results did not reach significance [25]. Alliesthetic changes after DS have also been shown to be persist up 2 years post-operatively, with patients attaining negative hedonics three times faster than normal weight controls. The point of satiety was also earlier than pre-operative and controls, giving further evidence to long-term changes acquired following metabolic procedures [94].

Changes in the reward value of fatty foods have also been investigated. Visual analogue scales provide a form of standardisation when assessing the pleasantness of an induced taste. Similar to other taste domains, RYGB has also been found to induce a change in the pleasantness perceived at higher concentrations of fatty solutions which is not observed in normal calorie-restricted weight loss [79].

The development of functional MRI (fMRI) has allowed the functional measurement of brain activity. Using image-based stimuli in VSG patients, Holsen et al. showed that the use of fMRI is a better predictor of total weight loss than hormonal or behavioural predictors [95]. Moreover, fMRI studies have shown differences in the neural responses to high- and low-energy food stimuli pre- and post-RYGB. An assessment of the effect of verbal and visual cues of different food types on regional stimulation found a significantly reduced response within the mesolimbic pathways in post-surgical patients in response to high-energy food images, as well as a less pronounced decrease in activation by low-energy food stimuli [96]. Similarly, a decrease in overall activation in response to food stimuli following RYGB has been reported in comparison to obese controls and LAGB patients, regardless of calorie content though significantly lower in response to high-calorie images. Additionally, RYGB patients had lower levels of activation across the reward pathways in response to high-calorie food stimuli compared to similar low calorie stimuli [97]. These changes in reward responses have helped explain the decreased pleasure noted by some patients post-surgically. fMRI studies have been used to measure taste responses to direct taste stimuli. Wang et al. used a taste delivery system to provide sweet and salty taste stimuli to subjects undergoing fMRI to measure perceived and biological hedonic responses. As expected, following pre-operatively, patients showed activation within the reward system and gustatory cortices in response to sweet stimuli, which decreased within the orbitofrontal and prefrontal cortices post-surgically. At 1 year post-operatively, there was a significantly weaker response in the left prefrontal cortex. Additionally, RYGB patients were found to have increased response in both the reward and gustatory cortices in response to salty stimuli, consistent with self-reported changes noted. While these results are consistent with previous reports, control subjects also showed a similar decrease in activation on the second visit in response to sweet taste, suggesting that habituation may be a confounding factor [98]. Similarly, Gershfeld et al. used fMRI to observe regional activation in patients undergoing LAGB adjustment. Deflation resulted in an increased desire for milkshake and a greater hedonic response than those who underwent band tightening. Tightening of the cuff resulted in activation of the emotional pathways in response to taste as opposed to a greater hedonic response [99].

## Conclusion

**Fig. 2:**
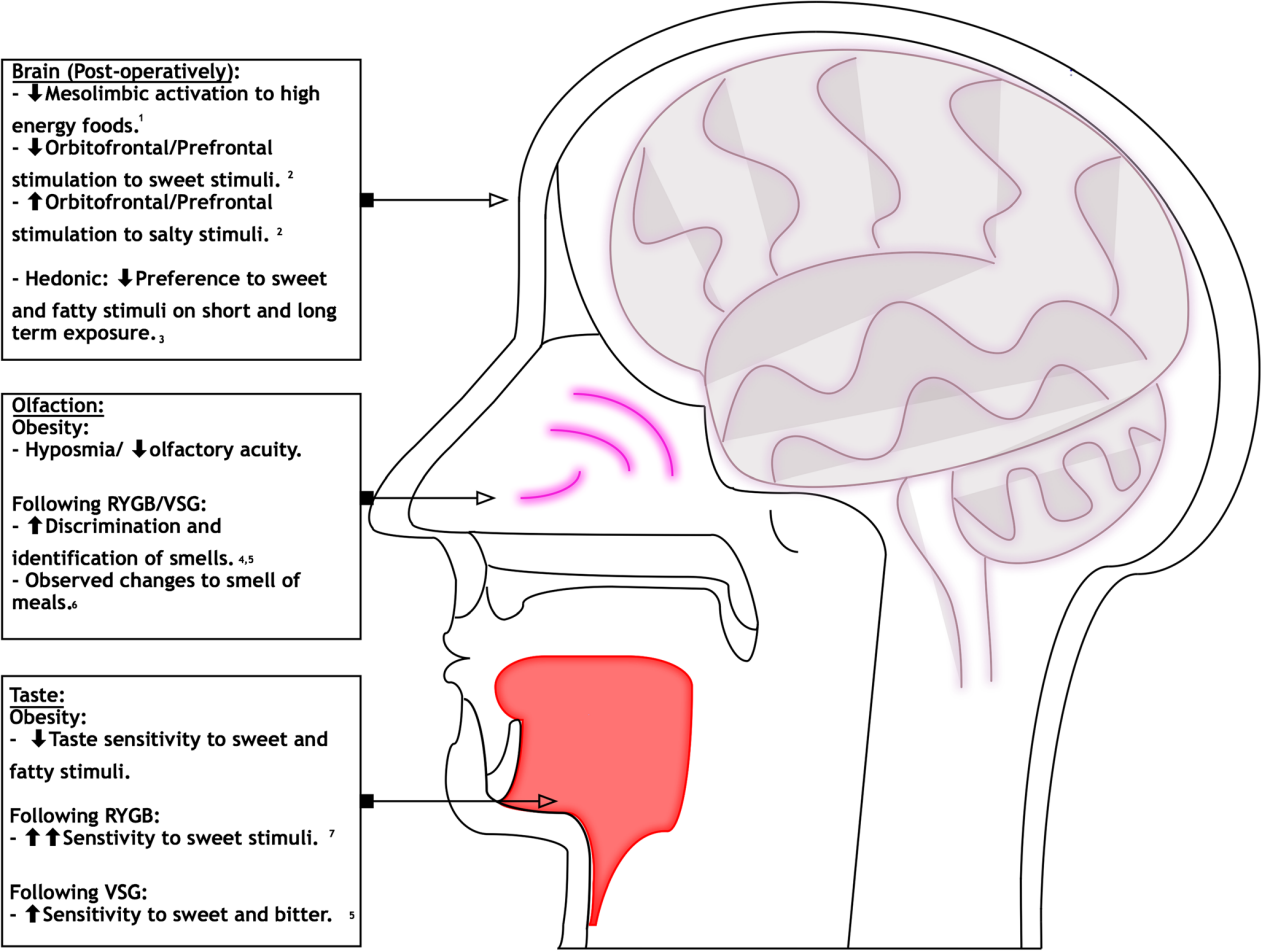
Schematic of changes in sensory organs in response to taste and smell following bariatric procedures

In conclusion, this study has found evidence which supports that reported changes in taste sensitivity and its relation to food preference may be partly due to intrinsic changes within the gustatory and olfactory systems following bariatric procedures (Fig. 2). Changes include an increase in sensitivity to sweet and fatty taste stimuli and a decrease in preference to sweet-tasting stimuli, as well as an increase in smell acuity. These changes may contribute to the long-term maintenance of weight loss post-operatively. Further research into underlying mechanisms may wish to look into quantifying patients who experience gustatory changes and identifying potential genetic and environmental factors which may facilitate these changes. Greater understanding into gustatory inputs in obesity and weight loss may provide an effective adjunct in the quest to find an effective medical treatment for obesity.

## Electronic supplementary material

Supplementary Table 1 (DOCX 12 kb)
