# Supplementary material for: Taste Changes after Bariatric Surgery: a Systematic Review
Source: Obes Surg. 2018 Jul 31;28(10):3321–32. doi: 10.1007/s11695-018-3420-8 (PMC6153588; doi:10.1007/s11695-018-3420-8)
Supplement: Supplementary file 1 — (DOCX 12 kb) [file 11695_2018_3420_MOESM1_ESM.docx]

| Initial key word | Taste | Bariatric Surger(y/ies) |
| --- | --- | --- |
| Expanded vocabulary included in search | Taste Sensitivity | Gastric Bypass |
|  | Taste Perception | Roux-En-Y |
|  | Taste Threshold | Sleeve Gastrectomy |
|  | Taste Acuity | Gastroplasty |
|  | Taste Discrimination | Gastrectomy |
|  | Gustation | Gastric Band |
|  | Gustatory Sensitivity | RYGB |
|  | Gustatory Perception | LAGB |
|  | Gustatory Threshold | Duodenal Switch |
|  | Gustatory Acuity | Jejunoileal Bypass |
|  | Gustatory Discrimination | Metabolic Surger(y/ies) |
|  | Smell | Bypass Surgery |
|  | Olfaction | Bariatric Surgery[MeSH] |
|  | Olfactory Threshold | Gastric Bypass[MeSH] |
|  | Sensory Thresholds | Jejunoileal Bypass[MeSH] |
|  | Olfacotry Perception |  |
|  | Olfactory Acuity |  |
|  | Olfactory Discrimination |  |
|  | Taste[MeSH] |  |
|  | Taste Acuity[MeSH] |  |
|  | Taste Perception[MeSH] |  |
|  | Taste Sensitivity[MeSH] |  |
|  | Taste Discrimination[MeSH] |  |
|  | Taste Threshold[MeSH] |  |
|  | Smell[MeSH] |  |
|  | Olfaction[MeSH] |  |
|  | Olfactory Perception[MeSH] |  |
|  | Sensory Thresholds[MeSH] |  |
|  | Olfactory Pathways[MeSH] |  |

*Supplementary Table 1: A list of identified search terms, including MeSH Synonyms.*
